# Trend Analysis of Industry-Sponsored Research Funding and Research Productivity in Orthopaedic Surgery Using the Open Payments Database

**DOI:** 10.7759/cureus.67396

**Published:** 2024-08-21

**Authors:** Romir Parmar, Chase Irwin, Sailesh Tummala, Heather Menzer

**Affiliations:** 1 Department of Orthopedic Surgery, University of Arizona College of Medicine, Phoenix, USA; 2 Department of Biostatistics, University of Arizona College of Medicine, Phoenix, USA; 3 Department of Orthopedic Surgery, Mayo Clinic, Phoenix, USA; 4 Department of Orthopedic Surgery, Phoenix Children's Hospital, Phoenix, USA

**Keywords:** orthopedic surgeons, industry funding, h-index, open payments database, research payments

## Abstract

Introduction

Orthopedic surgery and industry work together in order to provide optimal patient care. The Open Payments Database (OPD), established in 2013, reports industry payments to physicians. This study analyzes the first five years of industry-sponsored research funding (ISRF) to orthopedic surgeons and examines research productivity's effect on ISRF.

Methods

The OPD was queried from 2014 to 2018 for research payments to orthopedic surgeons in the United States. H-indices and publication volume were queried using the Scopus database. The research payments were sub-categorized to surgeons in teaching hospitals, registered clinical trials, preclinical research, and domestic.

Results

Between 2014 and 2018, a total of $202.74 million in ISRF was made to 1718 orthopedic surgeons. The proportion of research payments associated with a registered clinical trial significantly increased from 9.62% of payments in 2014 to 42.19% of payments in 2018 (p=0.002). Zimmer Biomet Holdings, Inc. ($20.77 million) contributed the largest value of payments to the greatest number of orthopedic surgeons (n=337). The total value of research payments increased by $3855 for every five-unit increase of a surgeon's H-index (p<0.001) and $762 for every five additional publications (p<0.001).

Conclusion

Orthopedic surgeons affiliated with a teaching hospital or clinical trial receive more ISRF. There may be a relationship between research productivity and ISRF.

## Introduction

The interdependent relationship between orthopedic surgeons and medical device industries is fundamental to the field, given the volume of implants used and the drive for innovation. Orthopedic surgery relies upon the development of new technologies in order to continually enhance patient outcomes [1]. Given this close relationship, however, financial conflicts of interest (COIs) between physicians and industry have been increasingly scrutinized in the last two decades [2]. Particularly, these disclosures are important to highlight due to unethical financial relationships in the past, such as a potential for monetary influence from industry on surgeons despite inappropriate treatment methods. Considering this, the majority of patients agreed that financial COIs be disclosed to them [3].

Consequently, prior to 2010, there were multiple legislative efforts to shine a light on physician-industry financial relationships, but it was difficult for these disclosure laws to achieve complete transparency [4]. There was also an attempt by certain industries to make their own payment data publicly accessible, but there were many inconsistencies in methods of doing so [5]. Finally, the Physician Payment Sunshine Act (PPSA) was passed under the Affordable Care Act in 2010, requiring the disclosure of industry payments or other transfers of value to physicians through the Open Payments Database (OPD) [6]. The OPD is made up of three categories of payments: 1) general payments, 2) research payments, and 3) physician ownership investments [7]. Research payments include direct physician compensation, study coordination, and implementation funding, or study participant payments.

Prior studies have evaluated the OPD to assess industry payment to orthopedic surgeons; however, only a few have investigated trends over time, including one study that demonstrated increases in both the number and value of industry payments to orthopedic surgeons over the time period of 2014 to 2018 [8]. Further, only a few studies have examined any other variables that may be associated with the number of payments made to orthopedic surgeons, such as academic influence. A prior study showed that academic productivity, as indicated by H-index and publication amount (Scopus, Elsevier Inc., Amsterdam, Netherlands), does not correlate well with industry research payments, but this study did not assess these relationships over time [9].

The present study aims to analyze the trends in industry-sponsored research funding (ISRF) made to orthopedic surgeons over the time period of 2014 to 2018, after the establishment of the OPD, and to evaluate funding specifically to orthopedic surgeons associated with teaching hospitals, for preclinical research, for registered clinical trials, and domestic payments as the primary outcome. Secondary outcomes include evaluating the correlation between the H-index, publication volume, and funding received, as well as reporting the amount of funding supplied by each manufacturer. The authors hypothesize that there would be an increase in research payments made to surgeons affiliated with teaching hospitals, and there would be a greater amount of ISRF to surgeons with more research productivity, as indicated by their H-index and publication volume.

## Materials and methods

Data on orthopedic surgeons

A retrospective database study was conducted in order to identify orthopedic surgeons who received OPD research payments between 2014 and 2018 in the Centers for Medicare and Medicaid Services (CMS) database. This time period was chosen in order to examine the preliminary trends seen in the OPD. Although the OPD was established in 2013, this year was excluded due to an incomplete year of data. Orthopedic surgeons were defined as Primary Investigators who indicated their specialty as Orthopedic Surgery on the OPD payment record. All research payments were adjusted for inflation by applying the dollar amount of each payment to the Consumer Price Index Inflation Calculator for January 2019. This time point was used to ensure that it encompassed the study period without going too far into the future. Payments towards orthopedic surgeons affiliated with teaching hospitals are those that had a Teaching Hospital listed on the OPD payment record. Payments for Registered Clinical Trials are those that had an associated ClinicalTrials National Clinical Trial (NCT) number on the payment. Payments were considered for preclinical research if the OPD payment record indicated preclinical research. If the manufacturer making the research payment was in the United States, it was considered a domestic payment. Fellowship training, subspecialty, and years of practice were determined by exploring each surgeon's profile on institutional websites or Doximity (Doximity INC, San Francisco, California) [10]. Orthopedic surgeons' H-index and number of publications for each year were extracted by searching each surgeon's research profile on the Scopus database (Elsevier Inc, Amsterdam, Netherlands) [11].

Statistical measures

For our primary analysis, we reported both the number of research payments and total inflation-adjusted US dollars paid to orthopedic surgeons stratified by year of payment. We also reported the proportion of total research payments made to orthopedic surgeons for each given year. 

For our subgroup analyses, we reported the number of orthopedic research payments and total inflation-adjusted US dollars made to orthopedic surgeons that were associated with the following: teaching hospitals, preclinical research, registered clinical trials, and domestic companies stratified by year of payment. The Cochran-Armitage test for trend was used to evaluate whether a statistically significant trend in the number of orthopedic research payments occurred from 2014 to 2018 for each designation. We evaluated trends in inflation-adjusted payments for each designation by fitting simple linear regression models with the proportion of total US dollars for the given year as our dependent variable and the year of payment as our independent variable. We reported the p-value of our estimated beta coefficient. 

Next we reported the top ten suppliers providing orthopedic research payments across 2014 - 2018. For each manufacturer, we reported the number of orthopedic surgeons receiving payments, the total inflation-adjusted dollar amount, and the proportion of total US dollars received by manufacturers. For our secondary analysis, we evaluated the impact of the H-index score and number of publications on total US dollars received by fitting mixed linear regression models with individual orthopedic surgeons treated as fixed effects. We reported the beta coefficient point estimate and its corresponding 95% confidence intervals and p-values for a five-unit increase for both predictors. An alpha level of 0.05 was used to determine statistical significance and all analyses were performed using SAS version 9.4 (SAS Institute Inc, Cary, North Carolina). 

## Results

**Figure 1:**
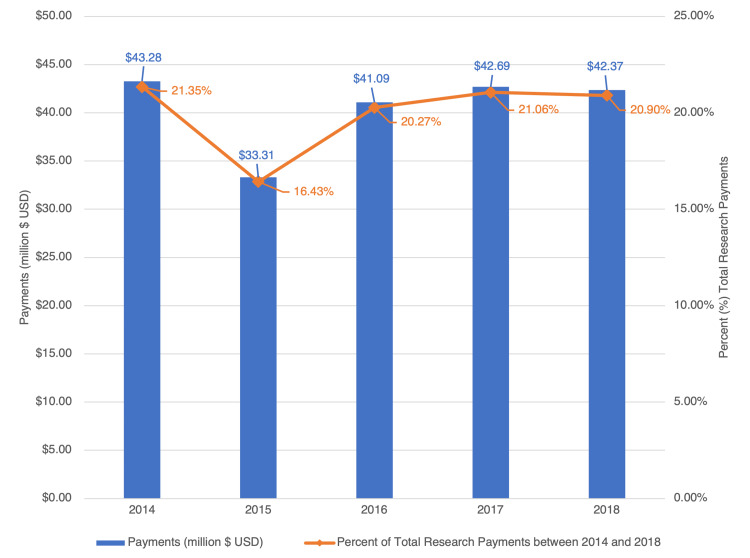
Research payments to orthopedic surgeons from 2014 to 2018 Value of research payments per year (bar graph) as well as the corresponding proportion of total research payments the payments comprised each year (line graph)

Between 2014 and 2018, there was a total of $202.74 million in industry-sponsored research funding (ISRF) made to 1718 orthopedic surgeons, which comprised a total of 29,589 research payments (Figure 1, Table 1). The monetary value of payments was greatest in 2014 ($43.28 million) and smallest in 2015 ($33.31 million). The total number of payments was the greatest in 2014 (n=6499); it was the smallest in 2016 (n=5311). Out of the total ISRF to all specialties, the ISRF to orthopedic surgeons was $43.28 million (0.84%) in 2014 and $42.37 million (0.85%) in 2018. Thus, the proportion of ISRF made to orthopedic surgeons did not significantly increase (p=0.61) from 2014 to 2018.

**Table 1:** Research payments made to orthopedic surgeons from 2014 to 2018 ^a^Adjusted for inflation using the Consumer Price Index (CPI) from the US Bureau of Labor Statistics; ^b^Reported as millions of US dollars (USD) spent

Year	Number of payments	% of total number of payments	Total inflation adjusted^a^ US dollars	% of total sum USD	Share of total value of research payments
2014	6499	21.96%	$43.28	21.35%	0.84%
2015	5671	19.17%	$33.31	16.43%	0.66%
2016	5311	17.95%	$41.09	20.27%	0.78%
2017	6004	20.29%	$42.69	21.06%	0.81%
2018	6104	20.63%	$42.37	20.90%	0.85%
Sum	29,589		$202.74		

Of the 1718 orthopedic surgeons who received research payments, the average orthopedic surgeon received $118,009 in payments, while the median orthopedic surgeon received $1593 in total research payments. The distribution of research payments is right-skewed as the highest-funded orthopedic surgeon received $1.5 million, and the top 10% received at least $15,000 in research funding.

Types of research payments

**Figure 2:**
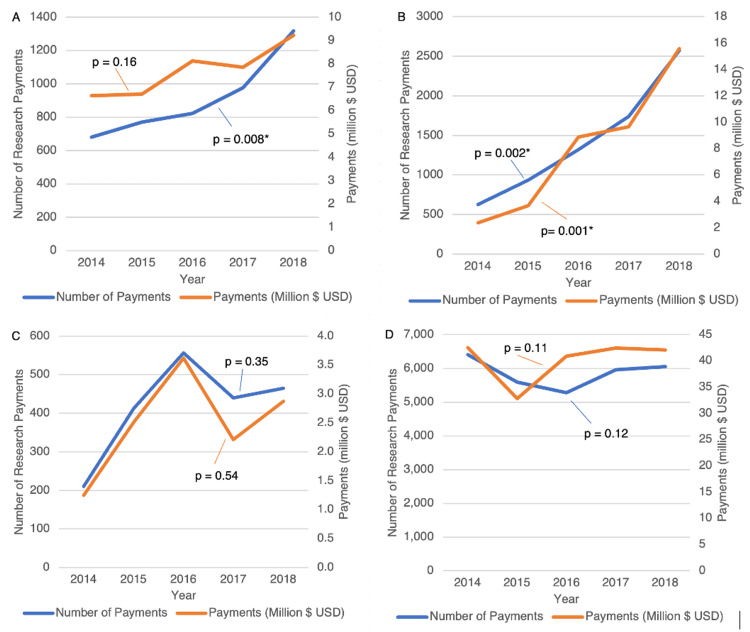
Types of research payments to orthopedic surgeons from 2014 to 2018 Number of different types of research payments and value of different types of research payments (in million $ USD) made to orthopedic surgeons associated with A) teaching hospitals, B) registered clinical trials, C) preclinical research, D) domestic countries per year. *Indicates statistical significance at a p<0.05

While orthopedic surgeons associated with teaching hospitals received $38.55 million (19.01%) of ISRF, those not associated with teaching hospitals received $164.19 million (80.99%). The proportion of research payments to orthopedic surgeons associated with teaching hospitals increased from 680 payments (10.46%) in 2014 to 1318 payments (21.59%) in 2018 in a steady and statistically significant upward trend (p=0.008) (Figure 2A). The monetary value of these payments increased from $6.64 million (15.34%) in 2014 to $9.23 million (21.78%) in 2018, but the trend was not statistically significant (p=0.16; Table 2).

**Table 2:** Trends in the characteristics of research payments to orthopedic surgeons *Indicates statistically significant result for alpha level = 0.05; ^a^Cochran-Armitage test for trend; ^b^Simple linear regression

Sub-type of research payment	Number of payments (% of total payments)	p-value^a^	Inflation-adjusted payments (% of total sum USD)	p-value^b^
Teaching hospital				
2014	680 (10.46)	0.008*	6.64 (15.34)	0.16
2015	770 (13.58)		6.70 (20.11)	
2016	823 (15.50)		8.12 (19.76)	
2017	977 (16.27)		7.86 (18.41)	
2018	1318 (21.59)		9.23 (21.78)	
Preclinical research				
2014	210 (3.23)	0.35	1.25 (2.89)	0.54
2015	413 (7.28)		2.51 (7.54)	
2016	556 (10.47)		3.62 (8.81)	
2017	440 (7.33)		2.21 (5.18)	
2018	465 (7.62)		2.87 (6.77)	
Registered clinical trial				
2014	625 (9.62)	0.002*	2.38 (5.50)	0.001*
2015	937 (16.52)		3.66 (10.99)	
2016	1318 (24.82)		8.89 (21.64)	
2017	1739 (28.96)		9.66 (22.63)	
2018	2575 (42.19)		15.58 (36.77)	
Domestic payments				
2014	6410 (98.63)	0.12	$42.51 (98.22)	0.11
2015	5593 (98.62)		$32.82 (98.53)	
2016	5280 (99.42)		$40.92 (99.59)	
2017	5959 (99.25)		$42.49 (99.53)	
2018	6059 (99.26)		$42.06 (99.27)	

Inflation-adjusted payments to orthopedic surgeons associated with a registered clinical trial totaled to $40.16 million (19.81%). The proportion of research payments associated with a registered clinical trial significantly increased from 625 payments (9.62%) in 2014 to 2575 payments (42.19%) of payments in 2018 (p=0.002). The proportion of the total value of payments made to registered clinical trials significantly increased from $2.38 million (5.50%) in 2014 to $15.58 million (36.77%) in 2018 (p=0.001) (Figure 2B)

Preclinical research received $12.47 million (6.15%) out of the total value of payments. In 2014, preclinical research payments were $1.25 million (2.89%), and in 2018, they were $2.87 million (6.77%) (p=0.54). The proportion of research payments for preclinical research did not significantly change from 210 payments (3.23%) in 2014 to 465 payments (7.62%) in 2018 (p=0.35; Figure 2C). 

Between 2014 and 2018, 99.04% of the total ISRF to orthopedic surgeons was made by domestic companies within the United States. The proportion of domestic payments from 2014 (n=6,410; 98.63%) to 2018 (n=6,059; 99.26%) did not increase significantly (p=0.12). The proportion of the total value of research payments made by United States companies also did not significantly change from $42.51 million (98.22%) in 2014 to $42.06 million (99.27%) in 2018 (p=0.11; Figure 2D).

Orthopedic suppliers and research payments

**Figure 3:**
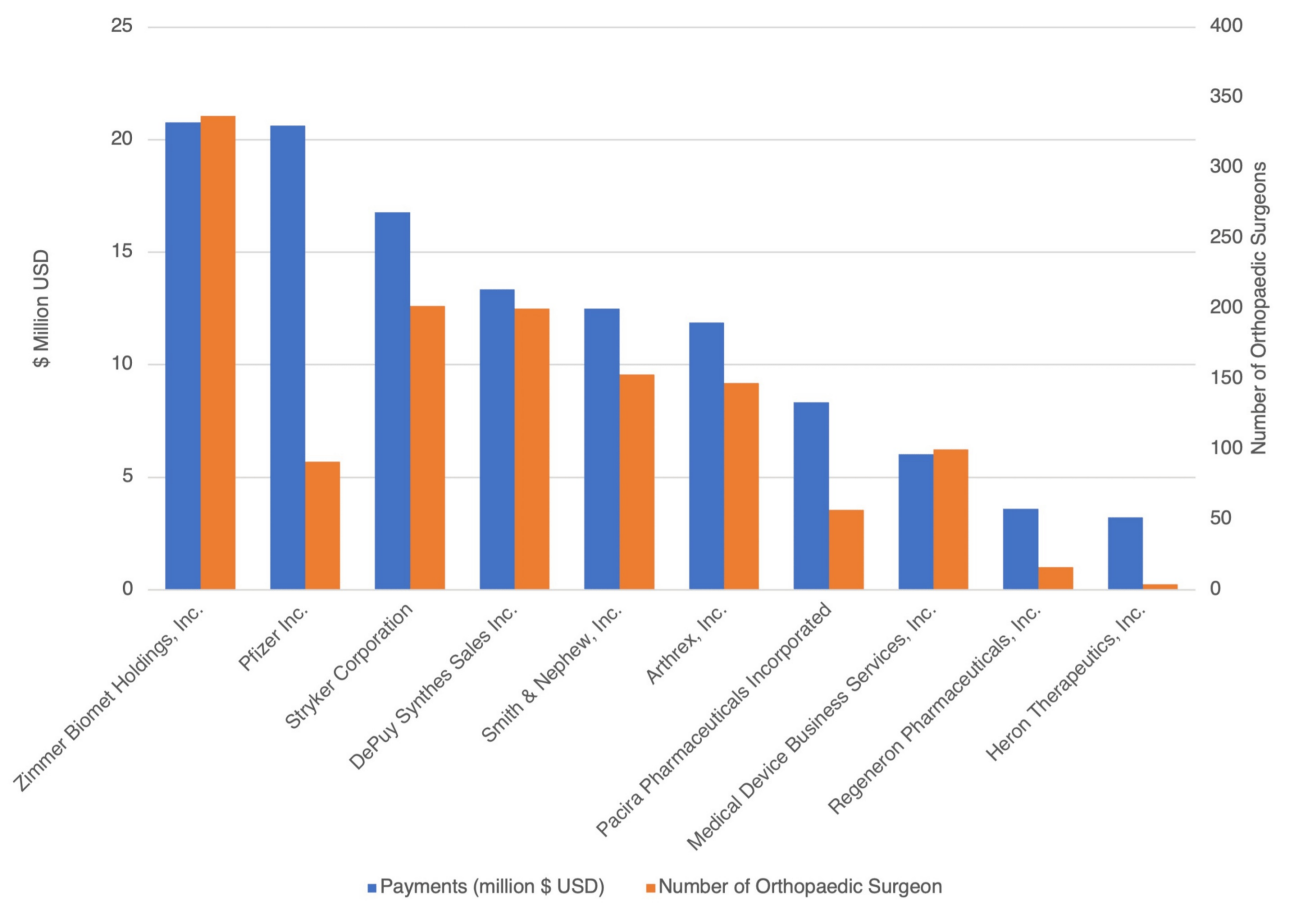
Top ten orthopedic surgery industry payors of research payments Total value of research payments in million $ USD from the top 10 industry payors (blue bars) as well as the number of orthopedic surgeons (orange bars) that received payments from that manufacturer between 2014 and 2018.

There were 60 different orthopedic suppliers that recorded at least $500,000 in ISRF to orthopedic surgeons. Of these, six provided at least $10 million in research payments (Figure 3). The suppliers who received the top 10 largest dollar amounts of research payments are shown in Table 3. Zimmer Biomet Holdings, Inc. ($20.77 million) contributed the largest value of payments to the greatest number of orthopedic surgeons (n=337). The Stryker Corporation contributed the third largest value of payments ($16.78 million), and reached the second-greatest number of orthopedic surgeons (n=202).

**Table 3:** Total value of research payments by manufacturer

Manufacturer making payment	Orthopedic surgeons receiving payments	Payments ($)	Percent (%)
Zimmer Biomet Holdings, Inc.	337	$20,772,260	11.18%
Pfizer Inc.	91	$20,625,017	11.10%
Stryker Corporation	202	$16,777,596	9.03%
DePuy Synthes Sales Inc.	200	$13,356,798	7.19%
Smith & Nephew, Inc.	153	$12,504,965	6.73%
Arthrex, Inc.	147	$11,892,975	6.40%
Pacira Pharmaceuticals Incorporated	57	$8,326,890	4.48%
Medical Device Business Services, Inc.	100	$6,019,952	3.24%
Regeneron Pharmaceuticals, Inc.	16	$3,599,533	1.94%
Heron Therapeutics, Inc.	4	$3,218,279	1.73%

H-Index and publication amount impact on ISRF

The total value of inflation-adjusted research payments significantly increased by an estimated $3855 for every five-unit increase of surgeon H-index (p<0.001; Table 4). The total value of inflation-adjusted research payments increased by an estimated $762 for every five additional publications (p<0.001; Table 4).

**Table 4:** Effect of H-index score and number of publications of total value of research payments received *Indicates statistically significant result for alpha level = 0.05; ^a^Beta coefficients were estimated for a five-point unit increase

	H-index score		Number of publications	
	Beta coefficent^a^ (95% CI)	p-value	Beta coefficient^a^ (95% CI)	p-value
Total $ value of research payments	3855 (1581 – 6129)*	<0.001*	762 (302 – 1221)*	0.001*

## Discussion

The financial relationship between orthopedic surgeons and the industry has become more transparent since the passing of the PPSA. The overall value of ISRF given to orthopedic surgeons did not significantly change across the time period of 2014 to 2018 (Figure 1). Though this is the case, there has been a substantial increase in the ISRF distributed to registered clinical trials from 2.38 million dollars in 2014 to 15.58 million dollars in 2018. Over this time period, an increase in the amount of research payments to orthopedic surgeons affiliated with teaching hospitals was also seen, despite the greater percentage of total research funding going towards those associated with non-teaching hospitals compared to teaching hospitals (19.01% vs. 89.55%; Figure 2). Orthopedic surgeons with a higher H-index and publication volume correlated with a greater amount of ISRF given to them (Figure 1). Out of a total of 60 orthopedic industry suppliers that recorded at least half a million dollars in ISRF, there were six suppliers that contributed $10 million in research payments each (Figure 3). In this study, the distribution of research payments is heavily right-skewed, as the average orthopedic surgeon received $118,009 in payments, while the median orthopedic surgeon received $1593 in total research payments over the study period.

ISRF for registered clinical trials experienced a marked surge over those five years. There could be multiple reasons for this, including increased regulation on disclosures. Since it is required by the PPSA that all payments, no matter how large or small, made by manufacturers to recipients be reported along, it is possible that this mandate itself could be associated with increased payments [12,13]. Another explanation for this can be attributed to the decrease in government funding for scientific research. While the National Institutes of Health (NIH) has a strong emphasis on translational research, orthopedic surgery as a whole receives a smaller number of NIH-sponsored research awards in the form of K awards when compared to other surgical specialties [14]. Specifically, from 2012 to 2018, there were ten K awards given to orthopedic surgeons, compared to eighteen K awards given in the prior six years [15]. Since orthopedic surgeons have historically received low amounts of NIH funding compared to other subspecialties, it could suggest that they are not applying for as many K awards due to reduced incentives [14]. Furthermore, the infrastructure to support basic science and clinical research in orthopedic research is challenging to develop [16]. Given this decline seen in government-sponsored research, there could be a greater push towards industries creating a closer partnership with academic hospitals to fund registered clinical trials. In this study, a large majority of the ISRF was made to orthopedic surgeons not affiliated with teaching hospitals. While it has historically been known that academic surgeons are more likely to be research-focused, this new finding may allow private and community surgeons to engage in research via industry sponsorship. On the other hand, ISRF may not be as prevalent in the teaching hospital setting due to various institutional guidelines and the higher likelihood of receiving government funding. Despite this, the number of payments towards orthopedic surgeons associated with teaching hospitals was found to significantly trend upwards directly after the establishment of the OPD and payment transparency. While it is difficult to tell if this increase is due to the beginning of payment reporting, it could indicate an association.

COI disclosure policies have become increasingly stringent since the initiation of the Physician Payment Sunshine Act in 2013 [17]. Notably, there has been an emphasis on ensuring orthopedic surgeons are transparent about ISRF when publishing in reputable journals. Working towards this goal, 18 orthopedic journals use the common International Committee of Medical Journal Editors (ICMJE) form on authors' COI disclosures. This increased the focus on accurate COI disclosure policies, but at the time of the study, there was not an agreement regarding editors' COIs.

Orthopedic surgery is a field where it is critical that appropriate relationships are maintained with manufacturers due to the necessity of surgical instrumentation and to ensure ongoing innovation to benefit patient care [1]. Zimmer Biomet Holdings, Inc. and Pfizer Inc. were the largest industry funders at $20.77 million and $20.63 million in payments, respectively. Zimmer Biomet Holdings Inc. also provided payments to the largest number of orthopedic surgeons at 337. Although Pfizer, Inc. provided payments to a smaller number of surgeons, they were able to pay greater sums of payments at an average of $226,649 per surgeon. In comparison to 2015, by the end of 2019, four of the top five orthopedic industry companies made general payment increases to surgeons [13]. This is supportive of this study's finding that the top six manufacturers recorded at least $10 million in payments, which demonstrates a growing partnership.

Orthopedic surgeons who are more productive in publishing research were correlated with receiving a larger amount of ISRF (Table 1). Although this does not establish a causative relationship, there is a link to those who have higher H-indices and amounts of publications when it comes to industry support. Previous studies have investigated the effect of academic influence on ISRF. Buerba et al. found that surgeons who received ISRF demonstrated higher average H-index and publication amounts than those without funding. Furthermore, they also noted weak correlations between the amount of ISRF and H-index and the number of publications [9]. These findings are consistent with our study, and they demonstrate how research productivity can have an impact on support received from the orthopedic industry partners. Further, it has been shown that orthopedic surgery departments with a higher H-index and M-index (H-index per year of research activity since first publication) are more likely to have a higher academic ranking [18]. This is an important aspect regarding ISRF to entire departments, as academic surgeons typically receive larger industry payments, specifically with regard to research [19].

Compared to other specialties in medicine, the proportion of ISRF to orthopedic surgeons is relatively low. Over the time period from 2014 to 2018, orthopedic surgeons have received less than 0.9% of ISRF. Despite this, funded orthopedic surgeons were found to have the highest impact per $100,000 of NIH grant funds they received. This impact was calculated based on the number of grant awards (R01s) received and the amount of funding received [20]. This was in comparison to other surgical specialties, including general surgery, urology, vascular surgery, and trauma surgery. Considering the efficient utilization of research funds by orthopedic surgery, a higher allocation of funding could be warranted. Prior research has suggested that larger payments to orthopedic surgeons are an independent predictor of research productivity [21]. In light of this, a strong surgeon-industry relationship could result in increased research funding, which paves an avenue to positively influence patient care through innovative technologies [22].

This study offers novel information about the specific types of research funding that orthopedic surgeons have received since the establishment of the OPD. The preliminary research payment trends in the OPD are described here, which allows future studies to relate future payment details to the initial data. Significantly, orthopedic surgery and the industry work mutually to improve outcomes. Although this may have initially led to some bias, the PPSA has been shown to increase transparency between the two sides and ultimately works towards lessening patient concerns about COIs. Further, it is notable to recognize the importance of research productivity and registered clinical trials for greater industry support.

Limitations

This study has several limitations. First, the trend analysis ended in 2018, so we are uncertain if the trends noted have remained consistent to the present day. Particularly, it would be interesting to evaluate if there was a sustained yearly increase in the amount of funding for registered clinical trials. While this study's analysis does not include present data, the main purpose of this study was to assess research payment trends immediately after the establishment of the OPD in 2013. Other studies chose this same time period, from 2014 to 2018, for the purpose of assessing initial trends in the OPD from the CMS [8,9,13,23]. Second, when investigating the effect of the H-index and publication amount on the amount of ISRF, it is important to understand that these variables are monotonic in that they will either remain the same or increase in value from year to year. Since the data reported in this study is purely descriptive, it is not possible to establish causal relationships. There are many factors that could influence the increase in the amount of ISRF given to a particular surgeon, including existing industry partnerships, institutional affiliation, and other factors. Finally, it is important to note that the data extracted here utilized a small proportion of the total number of practicing orthopedic surgeons. Since this cohort was mainly focused on the orthopedic surgeons who received research payments, this could be generalized to the surgeons in that setting, but it would not be able to describe the trends that may occur outside of this setting.

## Conclusions

In summary, this study demonstrated that orthopedic surgeons receive substantial industry-sponsored research funding per the Open Payments Database. This is important to highlight, as it indicates a strong collaboration between industry and orthopedic surgery in order to help improve patient outcomes. Specifically, the present study found a significant increase in the amount of research payments associated with a registered clinical trial over the five-year period. Furthermore, those that are associated with teaching hospitals received large amounts of ISRF. Orthopedic surgeons in academic settings are more likely to be involved with registered clinical trials, and thus, the industry may more commonly attempt to build relationships with surgeons in this setting and fund their research. It was also shown that the value of research payments made to orthopedic surgeons increased as the surgeon's H-index or publication number increased. Thus, the research productivity of an orthopedic surgeon may have a role in the amount of ISRF given, indicating that there may be an extra incentive for orthopedic surgeons to be more involved in research for purposes of receiving monetary support from non-government sources. 
